# Language transfers in third language acquisition of Chinese by intermediate level German and English native speakers: evidence from a behavioral experiment online

**DOI:** 10.3389/fpsyg.2024.1358603

**Published:** 2024-03-22

**Authors:** Ziqi Wang, Caihua Xu

**Affiliations:** ^1^School of Foreign Languages, Renmin University of China, Beijing, China; ^2^School of International Chinese Language Education, Beijing Normal University, Beijing, China

**Keywords:** language transfers, third language acquisition of Chinese, behavioral experiment online, similarities of abstract structural properties, language proficiency levels, Chinese language teaching

## Abstract

The paper investigates language transfers in third language acquisition of Chinese by native German and English speakers at intermediate level. Subjects are divided into two groups and complete a Grammaticality Judgment and Correction Task through a behavioral experiment online. The results from multiple sources show that: (1) both L1 and L2 are sources of language transfers and the perceived crosslinguistic similarity of abstract structural properties serves as the main reason; (2) language transfers can be non-facilitative on L3 learning; (3) as L3 proficiency level improves, the less likely learners are to be affected by non-facilitative language transfers in L3 learning, but it may not disappear completely; (4) the background language with higher proficiency level is more likely to impose language transfers in L3 learning. The research suggests that language transfers in TLA are simultaneously regulated by a number of factors, such as similarities of abstract structural properties between background languages and L3, as well as language proficiency levels. At the end, we discuss the application of the results to Chinese language teaching.

## 1 Introduction

Third language acquisition (TLA) refers to the situation in which a learner is currently studying one or several languages in addition to their native language and second language (Jessner, 1999, pp. 201–209; Jordà and Safont, 2005). Given that interlanguage interactions in TLA are much more complex than that in second language acquisition (SLA), the influence of learners’ previous language knowledge on third language learning has become a focal research issue (Cai, 2020, pp. 1–8 + 42 + 112). Language transfers, also known as “crosslinguistic influence (CLI),” or “interlinguistic influence,” indicate the influence of the commonalities and differences between the target language and other acquired languages (Odlin, 1989). It is a phenomenon in which language learners express ideas with the help of the pronunciations, word meanings, structural rules or expression habits of background languages (Ellis, 1994). Therefore, the role of background languages in TLA as well as the cognitive processes of language transfers has aroused great research interests. However, although current TLA research encompasses more than 30 languages, which is mainly represented by Indo-European languages, we have not seen many studies focusing on the transfers that cross different language families, especially language transfer studies on TLA of Chinese (Duan, 2020, pp. 119–127). Since the process of globalization has led to closer ties between countries, and the demand of multilingualism is thus growing, China is now playing an increasingly important role in the international arena. By the time of the global outbreak of the New Crown epidemic, China had become the largest Asian destination country for international students (Chinese Ministry of Education, 2019). Therefore, research on learning and teaching Chinese as a third language is also of great significance in practice.

Transfer is a psychological concept, which arose from behaviorist psychology. Behaviorism believes that people’s behavior can be explained by Stimulus- Response and that language is a type of behavior. Specifically, people respond when they are faced with a stimulus. If the response gets the desired result, then it is reinforced. Habits are formed after multiple reinforcements, and old habits influence the formation of new ones. When there are similarities between the two, the old habit will have a positive or negative influence on the new one, which is called “positive transfer” or “negative transfer.” In Selinker (1969), a famous American linguist, proved the existence of language transfers through seven experiments in his paper *Language Transfer*, which is the earliest empirical research in this field. Generally speaking, the development of language transfer research can be divided into four periods.

The first period was about in the 1950s and 1960s. Along with the flourishing of structuralism and behaviorism, language transfers became the basis for the predictions and explanations of the Contrastive Analysis Hypothesis, which considers the ultimate goal of language teaching to be the formation of a habit. The CAH equates language “differences” with learning “difficulties,” predicting possible errors in the learning process by analyzing differences between languages. The second period was in the 1960s and 1970s when many scholars found that some learners’ errors could not be explained by the CAH. During this period, the Errors Analysis Theory was put forward to explain the types of learner errors from the perspective of target languages, so as to get rid of the over-reliance on native language interference. However the main trend of second language acquisition research has turned to Chomsky’s universal grammatical framework, and the Markedness Theory began to be associated with language transfers. Language components are classified as marked or unmarked according to whether they have certain syntactic features. Differences between languages do not necessarily contribute to language transfers. Instead, the markedness serves as the determinant. In the third period, from the late 1970s to the 1990s, with the deepening of research, many scholars came to realize that language transfers had a significant impact on second language acquisition. Universal grammatical access and the initial state of the interlanguage became the focus of research in the field of second language acquisition, represented by the Full Transfer-, Full Access-, and the Minimal Trees Hypothesis. In the fourth period, from the 1990s to the present, based on the development of cognitive linguistics and psychology, the research on language transfers has been advanced to the cognitive level. Issues such as semantic transfers and pragmatic transfers began to draw people’s attention, focusing on influencing factors, sources and properties of transfers. Since TLA research is developing rapidly, language transfers in TLA have also become one of the hot topics in the past decades, producing a relatively rich set of results in various perspectives including influencing factors, sources and the modes of transfers.

According to the research, the influencing factors of language transfers in TLA can be summarized into two categories: language factors and learner factors. Language factors mainly include Language Distance or Psycho-language Distance, Second language status, Correctness perception, Foreign Language Aggregation, etc. Language distance, which is influenced by language family affiliation or geographical proximity, is considered by many scholars to be the main reason for language transfers. For example, Foote (2009) pointed out in his study that language distance is a decisive factor affecting facilitative transfer in TLA. Ringbom (2006) confirmed this idea when he found that native speakers of Swedish, which belongs to the same Germanic language group as English, are more likely to impose language transfers in English learning than Finnish native speakers. Psycho-language distance also affects language transfers, highlighting the learner’s subjective perception of languages, influenced by the learner’s meta-consciousness. Hammarberg (2009) argued that psycho-language distance is more accurate than objective language distance. The concept of Foreign Language Effect was introduced to describe the effect of a second language on a third language (Forsyth, 2014). Williams and Hammarberg (1998) proposed the concept of Second Language Status, which indicates that learners are more willing to activate the second language in TLA, inhibiting their mother tongues’ influence. De Angelis (2005) proposed the concepts of Correctness Perception and Foreign Language Aggregation to complement the idea of Second Language Status. Correctness perception indicates that learners perceive language transfers in TLA from their mother tongue as wrong and prefer to transfer from non-native languages, while the Foreign Language Aggregation refers to the cognitive system by which learners integrate non-native language knowledge.

Learner factors mainly include factors of learners’ language proficiency, meta-linguistic awareness, cognitive ability, learning strategies, age and so on. It has been found that the learners’ language proficiency levels of both the target language and the background languages have an important influence on language transfers in TLA. Navés et al. (2005) found that L3 English learners with low L3 proficiency borrowed more vocabulary from background languages compared to high-level learners. Hammarberg (2001) and Ringbom (2001) found that for learners with higher L2 language proficiency, L2 imposed greater effect on L3 learning. Conversely, the native language dominated language transfers. Other findings suppose that factors such as learners’ meta-linguistic awareness, development of cognitive skills and learning strategies, and age may likewise influence language transfers in TLA. Meta-linguistic awareness refers to the learners’ ability to consciously understand linguistic rules and monitor linguistic activities. Cenoz (2001) found that higher-aged learners were more prone to transfers, probably because learners’ meta-linguistic awareness and other factors develop as they grow older and accumulate learning experience. He further suggested that L3 learners have developed a higher level of meta-linguistic awareness, cognitive abilities and a wider range of learning strategies based on prior experience (Cenoz, 2013). In addition, learner’s educational background and language environment may also be influential factors in language transfers.

The discussion of the sources and properties of transfers is currently represented by the following research findings: (1) the L1 Factor Hypothesis and The Privileged L1 Transfer Hypothesis (Jin, 2009; Ranong and Leung, 2009; Hermas, 2010) suggest that the mother tongue is the psychologically preferred source of transfers. (2) The L2 Status Factor Model (Williams and Hammarberg, 1998; Bardel and Falk, 2007) suggests that the second language is more influential in early stages of language development. (3) The Cumulative Enhancement Model (Shanon, 1991; Williams and Hammarberg, 1998), the Typological Primacy Model (Rothman, 2011), the Linguistic Proximity Model (Westergaard et al., 2017), and the Scalpel Model (Slabakova, 2017) suggest that both the first and second language are sources of transfers. However, while CEM argues that language acquisition is cumulative and prior language learning experiences play a facilitative or neutral role in subsequent language learning processes, TPM, LPM, and SM recognize the existence of non-facilitative language transfers. In addition, TPM believes that language transfers are complete early in L3 interlanguage development, which means that they will only occur in one of the background languages that is most typologically similar to the target language. Learners, based on perceptions, would transfer the syntactic properties from a typologically similar background language on a large scale into the initial stages of TLA. LPM and SM hold different opinions that the perceived similarity of abstract structural properties between languages is the main reason for facilitating language transfers, rather than the similarity of language types.

In general, both language and learner factors are closely linked and thus work together. As to who dominates the language transfers, scholars have not come to a conclusion yet because it is difficult to isolate one of the factors and measure its influence. Moreover, the current research on TLA is mostly conducted based on evidence from Indo-European languages, with few studies focusing on Chinese as L3, so that the experimental data are not comprehensive and convincing enough to reach on a consensus and finish the discussion on the source and properties of language transfers.

In terms of research methodology, previous research on cross-linguistic influences in TLA could be summarized as following: comparative analysis, experiments, and longitudinal case study. Among them, the contrastive analysis method is the most traditional and commonly used research method, which allows for a more in-depth portrayal and a deeper understanding of structural properties by comparing different languages synchronically in order to reveal their similarities and differences. Most of the current mainstream language transfers studies have adopted the comparative analysis method in their experiment designs, including the CEM, TPM and LPM we mentioned above. For example, Jin (2009) examined the sources and properties of cross-linguistic influences based on output of L3 learners through comparative analysis. The study asked 40 L3 Norwegian learners (who had never learned Norwegian before arriving) with L1 Chinese and L2 English to finish a Grammaticality Judgment and Correction Task (GJCT) on “null objects,” while 14 native Norwegian speakers were set up as control group. Since the sentence structure of Chinese allows null objects, while English and Norwegian require a pronoun or noun phrase in object position, the authors conducted an experimental design accordingly, and found the strong influence of L1.

The experimental method mainly refers to online experiments of psycholinguistics and neurolinguistics, including behavioral, eye-tracking, and ERP experiments, etc., mostly examining unconscious language processing, aiming at exploring the cognitive processes of different languages. For example, Jin (2018) explored the activation mechanism of L2 orthography in L3 processing with the help of eye-tracking technology.

The longitudinal case study tracks learners’ L3 development trajectories over time through interviews, questionnaires, diaries, and writings. For example, based on Dynamic System Theory, Feng et al. (2022) investigated the developmental trajectories of syntactic complexity indexes in English writing among L3 English learners whose L1 were, respectively, Uyghur, Kazakh, and Mongolian with L2 Chinese.

Although it is said not convincing enough to use behaviorist concepts to explain the impact of CLI on foreign language learning and use, it makes considerable sense from a cognitive perspective to assume that learners in principle make use of any prior linguistic knowledge as “input” to the creative construction process (Faerch and Kasper, 1987). The learner’s background language is an important source of knowledge for TLA. However, we need to be clear about the “principle” here, which is one of the important aims of this study.

Therefore, based on the literature review, since most previous studies are offline, conducted based on questionnaires and corpora, this study conduct an online behavioral experiment drawing on research methods commonly used in psychological and neuroscience, combined with traditional comparative analysis, and uses E-prime as an experimental tool to investigate the language transfers in third language acquisition of Chinese by native German and English speakers at intermediate level. A Grammaticality Judgment and Correction Task was adopted in which the subjects were first asked to make a judgment on the correctness of given Chinese sentences. If it is an incorrect sentence, they are subsequently asked to correct it. In this way, we are able to gain a clearer understanding of the reasons why they subjects made such judgments while analyzing experimental results. Key presses and response durations each time of the subjects were also recorded. We hope to get a more comprehensive and realistic conclusion through the mutual corroboration of data from multiple perspectives.

Specifically, we expect to figure out the sources, properties and modes of language transfers in TLA by following questions: (1) do all background languages serve as sources of language transfers in the TLA of Chinese by German and English native speakers? (2) Do language transfers occur only in one of the background languages and are completing early in L3 interlanguage development according to language typological similarity? (3) Do non-facilitative language transfers exist in TLA of Chinese by German and English native speakers? (4) How do proficiency levels of background languages and L3 affect language transfers in TLA of Chinese by German and English native speakers?

## 2 Materials and methods

In this study, we examined the language transfers in Chinese TLA by intermediate level German and English native speakers through a behavioral experiment online using Grammaticality Judgment and Correction Task. The experiment was conducted by a 2 (syntactic structures) × 2 (groups) mixed design. Two structures examined were Topic-Comment (TC) and VO/OV in subordinate clauses. Participants were divided into two groups according to their language backgrounds, namely groups of native German speakers (DE) and native English speakers (EN). Since the VO structure is the basic principle of sequence in Chinese, we believe that intermediate Chinese learners should have learnt this structure and have a reasonably high level of language proficiency. While the TC structure, on the other hand, has beyond the grammatical points that intermediate Chinese learners should master according to the HSK. Based on this, we artificially created a discrepancy in L3 proficiency levels for each subject group.

### 2.1 Participants

Participants of our experiment were 40 undergraduate students majoring in Chinese-related subjects form universities in Germany and the United Kingdom, with an average age of 20.5 years old. They were divided into two groups according to their language backgrounds, the German group (L1 German- L2 English- L3 Chinese) and the English group (L1 English- L2 German- L3 Chinese). Each group consisted of 20 students, half of whom are male and the other half are female. Participants were recruited on a voluntary basis, paid accordingly at the end of the experiment. They were first interviewed to obtain basic information about them, including their age, gender, language backgrounds, and Chinese language levels. All of the subjects were required to be right-handed, with normal vision or corrected vision, and had passed the HSK level 3 with a vocabulary of 600–1,200 words, being able to read and comprehend Chinese sentences within the above vocabulary range. In addition, L2 of these test takers should have reached the European Standard B1 level, either English of German native speakers, or German of English, with the ability of reading and discussing most of the topics in daily life.

### 2.2 Materials and experimental tools

Materials consist of 52 Chinese sentences, composed of two parts, examining the two syntactic structures TC (Topic-Comment) and VO/OV. The TC structure consists of a topic, a topic marker, and a comment (Xu, 2000). The topic refers to the object of the sentence, which is something or someone known to both the speaker and the listener. Topic markers include words such as “啊, 吧, 呢,” in spoken language and a comma “,” which can often be omitted in written language. The part that describes the topic is called the comment. The VO/OV structure indicates the front and back position of the verbs and objects. The average word count of the TC part is 10.5, with a minimum word count of 8 and a maximum word count of 12, while the average word count of the VO/OV part is 15.2, with a minimum word count of 13 and a maximum word count of 17. All vocabulary used is based on the Chinese Vocabulary and Character Proficiency Level Syllabus (《汉语水平词汇与汉字等级大纲》) and is controlled in the range of HSK level 3 to minimize the influence of word length and raw words on the experimental results. All sentences in TC and VO parts are correct conforming to Chinese grammar, while OV part consists of sentences do not conform to Chinese grammar.

Based on whether the topics are generated by shifting, the topic-comment structure can be divided into two categories: (1) Base-generated Topics and (2) Dangling Topics or Chinese-style Topics (Xu, 2000; Hu and Pan, 2009). The former refers to topics that are generated by shifting sentence constituents and have a syntactic gap, while the latter indicates that the topic is an additive to the whole sentence (Hu et al., 2018). The two categories include four subcategories each, for a total of eight types of Topic-Comment structured sentences, and this paper focuses only on the case in which the topic is a noun phrase. Among them, two types of TC structures are similar to the syntactic structure of German. In English, on the other hand, it is unlikely to see such kind of expressions. The reason is that Chinese is one of topic-prominent languages, allowing the topic to be placed at the beginning of a sentence, while for English, which is a subject-prominent language, it is grammatical that the subject should be placed in the first position of a sentence. Here there are two examples (1, 2).


(1) Chinese: *这本  书     我  很         喜欢*。
             This  book  I   very much  like.
    German:  *Das  Buch  mag   ich   sehr.*
             This book  like  I     very much.
    English: “I like this book very much.”

This Chinese sentence belongs to one subcategory of the Base-generated Topic structures, where the topic moves from the object position to the topic position and leaves trace in the object position.


(2) Chinese: *下午        中国人   喜欢   喝      茶。*
             Afternoon  Chinese  like  drink  tea.
    German:  *Nachmittags  trinken   die Chinesen Tee   gern.*
             Afternoon    drink     Chinese      tea   like.
    English: “Chinese like to drink tea in the afternoon.”

This Chinese sentence belongs to another subcategory of the Base-generated Topic structures, in which prepositional and directional phrases that express time and place remove the preposition or directional word and move to the topic position.

Due to the fact that there are similar sentence structural properties to the above Chinese TC structures in German and not in English, the TC section was divided into TC1 and TC2. TC1 consists of the two types of Chinese TC structures mentioned above, with six sentences in each category, and TC2 consists of the remaining six types of Chinese TC structures, with two sentences in each category. For TC2, there is no similarity of structural properties between either German and Chinese, nor English and Chinese, which is designed as a section of filler sentences. Considering that topic markers in TC structures may have an effect on the experimental results, half of the sentences in TC1 and TC2 were marked with the topic marker “,”.

Furthermore, the basic word order in both Chinese and English is SVO, which is still maintained in subordinate clauses. German, on the other hand, is more flexible in the position of the constituents except for the verb position. In subordinate clauses of German, verbs are always placed at the end of the sentence, showing the SOV structure. Here is one example (3).


(3) Chinese: *当   我的 爸爸    打开     电视  的时候，     我  在  看    书。*
             when my  father turn on  TV   the moment  I   be read  book.
    German:  *Wenn mein Vater  das Fernsehen anmacht, lese  ich  ein  Buch.*
             when my   father the TV        turn on  read  I    a    book.
    English: “When my father turns on the TV, I am reading a book.”

Based on the constituents of the subordinate clauses in the sentence, the subordinate clauses can be classified into three types: noun (subject, object, epithet, cognate), adjective (determiner) and adverbial (gerund) clauses. Therefore, in the 28 VO/OV structure sentences in this experiment, we divided the VO and OV structures into two sections. Both parts include 2 sentences each of noun subject clauses, noun object clauses, determiner clauses, temporal clauses, causative clauses, locative clauses, and conditional clauses, and half of the sentences are marked with the marker “,”. Sections of experimental materials are shown in Table 1.

**Table 1:** Sections of experimental materials.

Sections	Structures
TC1	2 selected subcategories of Topic-Comment structure
TC2	Other subcategories of Topic-Comment structure
VO	Subordinate clauses in VO sequence
OV	Subordinate clauses in OV sequence

E-prime2.0 was used to write the experimental program. The presenting order of 52 sentence for each participant were randomly generated by the software and presented only once. Five native Chinese speakers were first recruited to complete the experiments independently. Based on the results, we modified the ambiguous parts of the first draft of the materials. Then, five intermediate German and English Chinese learners (L1 German-L2 English-L3 Chinese or L1 English-L2 German-L3 Chinese) conducted the pre-experiment. We adjusted the vocabulary difficulty and sentence selections.

### 2.3 Experimental procedure and scoring

The experiment was a Grammaticality Judgment and Correction Task, which was conducted using computer E-prime 2.0 software. The subjects were asked to judge whether the Chinese sentences in the four sections TC1, TC2, VO, and OV were correct. If the sentences were grammatical, the subjects pressed J and entered J in the pop-up text box; if they were ungrammatical the subjects pressed F and corrected the sentences in the text box. There was no time limit for the experiment, and the subjects could think through their answer before responding. The software records the selected keystroke, the content of the modification and the reaction time of each keystroke. The reaction time was calculated from the presentation of the target sentence on the screen to the end of pressing the corresponding judgment key, and the text box content input time was not included in the reaction time. The specific experimental steps were as follows.

**Figure 1:**
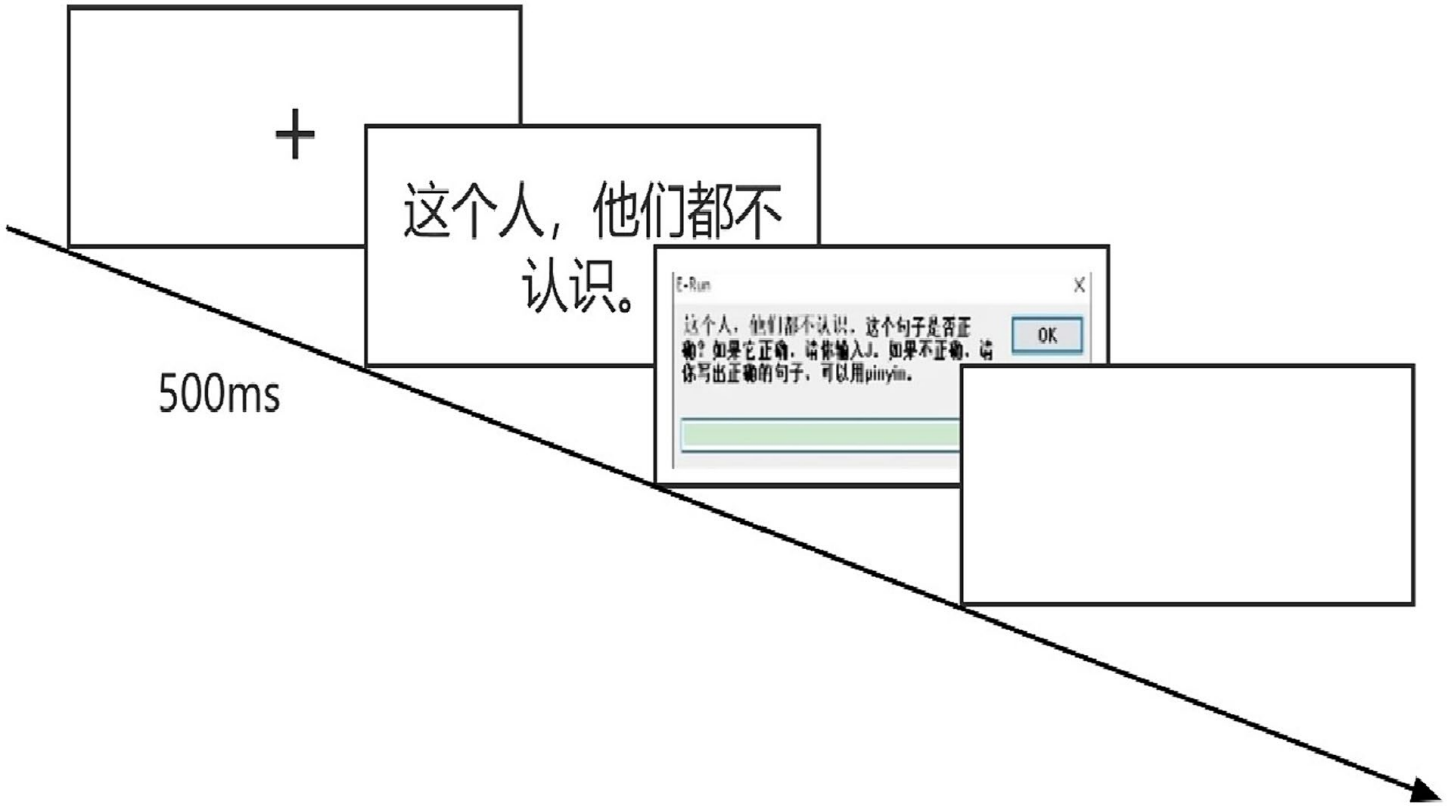
Experimental flow chart.

Step 1: Guidance reading. The subject was asked to read the instruction on the screen and press the required keys to proceed to the next step.
Step 2: Gaze point guidance. A black “+” mark appeared in the center of the screen for 500 ms, directing the subjects’ attention to the central position of the screen, indicating that the experiment was about to start.
Step 3: Practice session. Subjects completed an exercise consisting of 5 sentences to familiarize themselves with keystroke operations and dialog box text input. A random procedure was used to randomly present the stimulus material, one target sentence at a time, and the subjects were required to judge whether the sentences were correct or not. The content of the stimulus materials in the practice session was not related to the formal experimental session. After the practice session was completed, the subjects could choose to practice again or start the formal experiment.
Step 4: The formal experiment session. This was the same as that of the practice session, as the subjects read a total of 52 test sentences covering 4 sections in random order and made judgments and corrections. After they completed all of the questions, the software jumped to the acknowledgement page and ended the experiment. The experimental flow chart is shown in Figure 1.

The experimental score for each subject was calculated according to the following criteria: 2 points for each sentence.

Since all test items in TC and VO sections were grammatical Chinese sentences, subjects were awarded 2 points for correct judgment (by pressing J and typing J into the pop-up text box). If subjects thought the sentences were wrong (by pressing F and typing the corrected sentences into the pop-up text box), the scorer graded them according to the subject’s modified sentences. There were two cases: if the new sentence entered by the subject modified the syntactic structure examined by the test item (i.e., TC structure or VO structure), we considered that the subject took the given sentence as ungrammatical, and then no points ware given; if the subject modified other parts of the sentence other than the tested structure, then 1 point might be given at the discretion of the scorer.

As for OV section, all tested items were ungrammatical Chinese sentences and needed to be modified by the subjects. 1 point was be awarded for correct judgment (press F). Another 1 point was awarded for correctly modifying the given ungrammatical Chinese sentence with OV structure into a sentence with VO sequence.

Three native Chinese speakers independently scored the responses back-to-back, and they gave the final scores after discussing the discrepancies.

### 2.4 Data collection and processing

The scores and reaction times data were firstly collated. Then the mean scores, percent correct (results were retained to two decimal places) and mean reaction times were calculated for 4 different sections, TC1, TC2, VO, and OV. In order to investigate the main effects and possible interactions, the present study was analyzed by repeated measures ANOVA using SPSS 23 with mean score and mean reaction time as dependent variables and groups and structures as fixed factors, respectively. In addition, the results of the subjects’ sentence modifications were organized and categorized, the frequencies of each kind of modification types were counted, and a chi-square analysis was performed using SPSS 23.

## 3 Results

The mean score, percentage correct and mean response time data for each section for both groups of subjects are shown in Table 2.

**Table 2:** Mean scores, percent correct and mean reaction times of both groups.

Groups	Structures	Secondary categories	Mean scores (standard deviation)/Percent Correct	Average reaction time per sentence (ms) (standard deviation)
DE	TC	TC1	1.76 (0.65)/0.88	6,728 (5718.8)
		TC2	1.77(0.64)/0.88	6,582 (5059.2)
	VO/OV	VO	2(0.06)/1	6,011 (3933.52)
		OV	0.91 (0.97)/0.43	8,601 (7224.34)
EN	TC	TC1	1.54(0.84)/0.77	7,235 (5926.47)
		TC2	1.66 (0.75)/0.83	5,708 (5414.82)
	VO/OV	VO	1.99(0.12)/1	6,061 (4908.37)
		OV	1.33(0.92)/0.66	6,619 (3157.67)

As can be seen from the table, the German group scored higher in the TC section (*M* = 1.76, *SD* = 0.65) than the English group (*M* = 1.60, *SD* = 0.80), and the mean reaction time for processing the TC1 structure (*M* = 6727.81, *SD* = 5718.8) was shorter than that of the English group (*M* = 7235.03, *SD* = 5926.47). On the contrary, the English group scored higher in the VO/OV section (*M* = 1.66, *SD* = 0.73) than the German group (*M* = 1.45, *SD* = 0.88), and the reaction time for processing the OV structure was shorter (*M* = 6619.10, *SD* = 3157.67) than that of the German group (*M* = 8600.60, *SD* = 7224.34).

In the following, we interpret the experimental results from three perspectives: score, response time, and sentence correction results, respectively.

### 3.1 Scores

**Figure 2:**
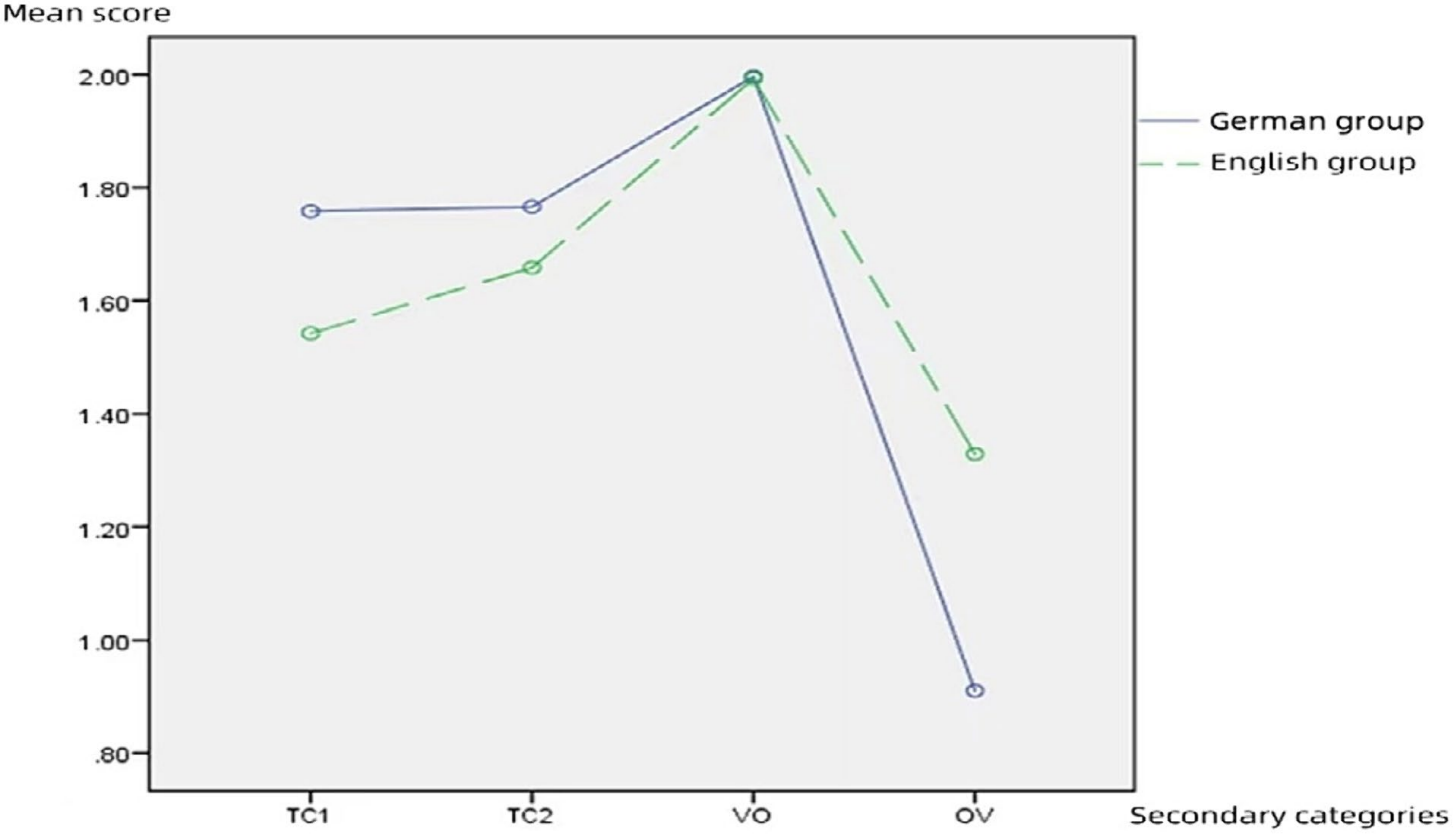
Mean scores of German and English groups.

Mean scores of German and English groups are shown in Figure 2.

The results of the multivariate test found significant differences in scores between 4 categories, *p* < 0.001, and an interaction between categories and subject groups, *p* = 0.01. The spherical test of significance was *p* < 0.001, and so subsequent analyses were carried out using the correction coefficients of the Greenhouse–Geisser method. The results of the within-subjects effect test were consistent with the results of the multivariate test, *F*_category_ = 60.044, *p* < 0.001, and *F*_category × group_ = 8.674, *p* < 0.001, both of which concluded that there was a significant difference in subjects’ scores by categories and that there was an interaction between the categories and subject groups. The results of the within-subjects comparison concluded that the changes in the scores of the different categories conformed to a linear relationship, *F* = 22.118, *p* < 0.001. The test of variance chi-square found that all *p*’s were greater than 0.05, suggesting that the data on the scores of the different categories were variance chi-square and were suitable for the analysis of variance (ANOVA). The between-subjects effect found that the two groups performed similarly in terms of scores. There was no significant difference, *F* = 0.138, *p* = 0.713. Given that there was an interaction between categories and subject groups, we proceeded to a simple effects analysis.

As can be seen from Table 3, in terms of scores, there was a significant difference between the two subject groups on TC1 and OV categories. No significant difference was found between the scores of the two groups on TC2 and VO sections.

**Table 3:** Simple effects analysis (Categories × Groups).

Categories	(I) Group	(J) Group	Significance
TC1	DE	EN	0.044
	EN	DE	0.044
TC2	DE	EN	0.333
	EN	DE	0.333
VO	DE	EN	0.657
	EN	DE	0.657
OV	DE	EN	0.004
	EN	DE	0.004

As can be seen from Table 4, for the German group, the differences in scores between the TC1 and VO/OV categories as well as the VO, OV sections with other categories were significant. For the English group, the differences in scores were significant for all two categories except for the difference between TC1 and OV as well as TC1 and TC2.

**Table 4:** Simple effects analysis (Groups × Categories).

Groups	(I) Categories	(J) Categories	Significance
DE	TC1	TC2	0.902
		VO	0.002
		OV	0.000
	TC2	TC1	0.902
		VO	0.004
		OV	0.000
	VO	TC1	0.002
		TC2	0.004
		OV	0.000
	OV	TC1	0.000
		TC2	0.000
		VO	0.000
EN	TC1	TC2	0.083
		VO	0.000
		OV	0.086
	TC2	TC1	0.083
		VO	0.000
		OV	0.009
	VO	TC1	0.000
		TC2	0.000
		OV	0.000
	OV	TC1	0.086
		TC2	0.009
		VO	0.000

In summary, the scoring data indicate that (1) the overall score differences between subject groups were not significant while the differences in scores between the different categories were significant. (2) Further analysis reveals the significant differences in the TC1 and OV sections between groups. The German group scores significantly higher than the English group in TC1, while the English group scores significantly higher in OV. (3) Both groups achieve their highest mean scores in the VO section, which was significantly different from the scores in the other categories. The least significant difference in scores was found between the subject groups in the VO category. (4) There was a significant difference in performance between the VO and OV categories.

### 3.2 Reaction time

Given that the original reaction time data showed a right skewed distribution, we performed a logarithmic transformation of the data to normalize the reaction time data with the following equation:


$$Y^{\prime }=\mathrm{lg}\;Y\text{.}$$

*Y* refers to the original reaction-time data and *Y*′ is the transformed data. Detailed data are shown in Table 5.

**Table 5:** Original data and transformed data at the time of reaction.

Groups	Structures	Secondary categories	Original data (standard deviation)	Transformed data (standard deviation)
DE	TC	TC1	6,728 (5718.8)	3.83 (0.32)
		TC2	6,582 (5059.2)	3.82 (0.29)
	VO/OV	VO	6,012 (3933.52)	3.78 (0.26)
		OV	8,601 (7224.34)	3.93 (0.29)
EN	TC	TC1	7,235 (5926.47)	3.86 (0.33)
		TC2	5,708 (5414.82)	3.76 (0.31)
	VO/OV	VO	6,061 (4908.37)	3.78 (0.28)
		OV	6,619 (3157.67)	3.82 (0.19)

A multivariate test of the transformed data revealed that there was a significant difference in subject response times between question categories, *p* = 0.048. Given that the significant result of the test of sphericity was *p* < 0.001, the subsequent analyses were conducted using the correction factor of the Greenhouse–Geisser method. The within-subjects effect test was consistent with the multivariate test, *F*_category_ = 3.510, *p* = 0.039, both of which showed that there was a significant difference in subject response times across the categories. The variance chi-square test found that all *p*’s were greater than 0.05 and therefore suitable for ANOVA. The between-subjects effect test showed that *F* = 0.209 and *p* = 0.650, indicating that the overall response duration was similar between the two subject groups and there was no significant difference in performance.

To conclude, we got the following results based on analysis of reaction time: (1) there were no significant differences in the response time data between the subject groups, and there were significant differences between the categories The German group took less time in the TC1 section, whereas the English group took less time in the OV section. (3) Both groups took shorter time in the VO section, and the difference between groups was not significant. The above results are consistent with the score data and corroborate each other.

### 3.3 Sentence correction

We firstly categorized the Chinese sentences produced by the subjects in the experiment, after which we conducted frequency counts for each mode.

The modes of correction by the subjects of Chinese sentences with TC structure could be summarized into three categories, which were referred to as Mode 1, Mode 2, and Mode 3, respectively. Mode 1 referred to the modification of topics, i.e., subjects removed the topic at the beginning of a sentence by moving or deleting it, or added to it so that it became a sentence component such as a determiner or a gerund. Here are two examples comparing the items with TC structures (Example 1a, 2a) and their modified sentences (Example 1b, 2b).


(1) a. *这个  人,    他们   都  不  认识。*
       this person they  all not know.
       “They do not know this person.”
    b. *他们  都   不  认识  这个   人。*
       they  all not know this  person.
       “They do not know this person.”


(2) a. *北京     很多   博物馆     是   免费的。*
       Beijing  many  museums   be  free.
       “Many museums in Beijing are free.”
    b. *很多   北京的      博物馆   是   免费的。*
       many  Beijing’s  museums  be  free.
       “Many Beijing’s museums are free.”

In Example 1, the modification has moved the topic “这个人 (this person)” at the beginning of the sentence to the object position of the sentence. In Example 2, many participants in the experiment have added a Chinese character “的” to the sentence and transformed the topic “北京 (Beijing)” into an attribute of museums.

Mode 2 referred to the modification of the overall structure of the sentence, i.e., modifying other components of the sentence instead of the modification of topics, such as the modification on subjects, orders of the words, passive forms, etc. Here is an example (3a, 3b).


(3) a. *那本   书    它   还    在  书包       里。*
       that   book it  still  be schoolbag  in.
       “That book is still in the schoolbag.”
    b. *那本  书    还     在  他的  书包       里。*
       that  book still  be  his  schoolbag in.
       “That book is still in his schoolbag.”

In Example 3, many participants have transformed the subject “它 (it)” into the attribute “他的 (his)” of the schoolbag and “那本书(that book)” became the subject after modification.

Mode 3 referred to the modification of the other components of the sentence, i.e., the addition, deletion or modification of the wording of the other components of the sentence without modifying the topic at the beginning of a sentence and the overall structure of the sentence. Here is an example (4a, 4b).


(4) a. *那只   猫  我 不会      忘记    它。*
       that  cat I  will not forget  it.
       “I will not forget the cat.”
    b. *那只  猫  我   永远     不会      忘记   它。*
       that cat I    forever will not forget it.
       “I will never forget the cat.”

In Example 4, there were some participants who added the word “never” to modify the predicate of the sentence to make the sentence sound more fluent.

As for the structure of VO/OV, the ways in which the subjects modified the sentences could also be classified into three categories. Mode 1 referred to the modification of the predicate-object sequence in subordinate clauses. Since VO is the correct order of Chinese subordinate clauses, sentences in OV sections are ungrammatical Chinese and we expected the participants to modify them. Here is an example (5a, 5b).


(5) a. *我  电影    看     的时候,      经常    会    吃    饼干。
        I   movie  watch  the moment  often  will  eat  cookies.
       “*When I movies watch, I often eat cookies.”
    b. *我 看     电影    的时候,      经常    会    吃    饼干。*
       I  watch  movie  the moment  often  will  eat  cookies.
       “When I watch movies, I often eat cookies.”

In Example 5, many participants were able to modify the sequence of OV to VO in the subordinate clauses.

Mode 2 referred to the modification of the overall structure of the sentence, i.e., instead of modifying the predicate-object order in the subordinate clauses, other components of the sentence such as the subject, the active-passive form, and the order of other parts of the sentence were modified. Here is an example (6a, 6b).


(6) a. *如果  爸爸   他     表扬了,  他  会    很      开心。
       if    father him   praise  he  will  very   happy.
       “*If the father him praises, he will be very happy.”
    b. 如果 他 被           爸爸     表扬了, 他   会    很   开心。
       if  he be(passive)  father  praise he   will very happy.
       “If he is praised by his father, he will be very happy.”

In Example 6, some subjects preferred not to adjust the VO order of the subordinate clause, choosing instead to change the sentence to the passive form.

Mode 3 referred to the modification of other components of the sentence, i.e., instead of modifying the sentence topic-statement structure and the overall structure of the sentence, additions, deletions, or modifications of wording have been made to other components of the sentence. Here is an example (7a, 7b).


(7) a. 那个  昨天      打破   窗户    的人   在哪?
       that yesterday break window person where.
       “Where is the yesterday window breaking person?”
    b. 昨天       打破  窗户    的那个 人     在哪?
       yesterday break window that  person where.
       “Where is the person who broke the window yesterday?”

In Example 7, some participants judged the sentence to be “wrong” and revised the position of the adjunct “那个(that).” This kind of modification did not change the TC or VO structure of the sentence but adjusted the other components to make the sentence sound correct.

Based on the above classification of the modifications produced by the German and English groups toward Chinese sentences of TC and VO/OV structures, the frequency of each mode of modifications was counted. The results are shown in the Table 6.

**Table 6:** Frequency statistics of sentence modification modes of both groups.

Groups	Secondary categories	Mode 1	Mode 2	Mode 3	Total
DE	TC1	29	0	0	29
	TC2	27	0	2	29
	VO	0	3	5	8
	Total	56	3	7	66
	OV	126	5	4	135
EN	TC1	45	5	1	51
	TC2	35	5	3	43
	VO	0	2	9	11
	Total	80	12	13	105
	OV	181	8	4	193

It should be noted that for TC & VO sections, since the experimental materials are grammatical Chinese sentences, modifying the sentences by “mode 1, 2 and 3” means that the subjects made wrong judgments, and thus the lower the frequency, the better the performance of the group. However, as for the OV section, since the OV sequence in subordinate clauses is ungrammatical in Chinese, the frequency of “Mode 1” indicates the frequency of subjects correctly modifying the sentence after correctly judging. Therefore, the higher the frequency of “Mode 1,” the better the performance of the subject group. After dividing the 4 categories into two parts, TC&VO and OV, we then conducted a chi-square analysis separately.

Observing the statistical results in the above Table 6, it is found that the modification frequency of the two subject groups in the VO category is much lower than that in the TC1 and TC2 parts. Since the test items in TC & VO categories were grammatical Chinese sentences, the lower modification frequency indicated the better performance. Therefore, we could intuitively see that the subjects performed best in the VO category. Besides, the German group perform better than the English group in the TC1 section while the English group perform better in the OV section, which is consistent with the results of the score and response time analyses.

The results of the chi-square test found that for the TC&VO part, the difference between the two subject groups was not significant either in terms of the total frequency of three categories for each mode (*X*^2^ = 2.68, *p* = 0.262) or in terms of the total frequency of use of the three modes for each category (*X*^2^ = 0.370, *p* = 0.831). As for the OV section, the difference between the two subject groups was significant in terms of the frequency of use of the three modes, *X*^2^ = 6.689, *p* = 0.035, which, as can be seen from the table, is mainly reflected in the difference in the frequency of Mode 1.

A further cross-sectional statistical analysis of each participant’s production in the four categories was conducted. We found that 5 subjects from the German group scored full marks in the TC1 section, while the rest of the subjects have used different modes to incorrectly modify the test items in TC. Besides, in the OV section, 2 subjects from the English group got full marks, while the rest of the subjects fail to correctly judge and modify the OV sequence of the given sentences. This finding also indicated that German group performed better in TC1 section, while the English group did better in the OV.

To summarize, the statistical analysis based on sentence modifications revealed the following results: (1) For the TC and VO sections where the test items were grammatical Chinese sentences, the performance of the subjects was similar between the groups, with no significant differences. For the OV section where the test items were ungrammatical Chinese sentences, the English group performed significantly better than the German group. (2) Among all the subjects, only a few subjects in the German group got all the scores in TC1, and only a few subjects in the English group got all the scores in the OV category, and most of the subjects made errors in both TC1 and OV categories. (3) Both groups performed best in the VO category. The above results were consistent with the findings from the score and response time data.

## 4 Discussion

Based on results from multiple data sources above, the following discussion is made.

### 4.1 Sources, properties, and modes of language transfers in TLA

Subjects in the experiment were asked to correct sentences when they thought the given Chinese sentences in the experiment were ungrammatical in order to visualize their mastery of the syntactic structures under examination and the reasons for their judgments. German has a relatively flexible linguistic framework, with the exception of the main clause in which the predicate verb is always in second place. The rest of the components have considerable flexibility. Placing the topic or emphasis at the beginning of the sentence and postponing the subject is a very common sentence structure in German. On the contrary, this type of framework is ungrammatical in English. Hence, two types of Chinese TC structure sentences with similar structure properties in German were selected for the design of the experimental material for the TC1 section. All of them were grammatical Chinese sentences. The statistics of the sentence modification results revealed that more than half of the subjects in both groups made wrong judgment in the TC1 section. Of these, all the German group subjects who made incorrect judgments used Mode 1. They tried to correct the sentences by moving or deleting the topics at the beginning of the sentence to ensure the prominence of the subject at the beginning of the sentence, which is consistent with the grammatical rules of English. Therefore, we believed that subjects of German group were affected by the non-facilitative transfers of English.

The experimental results in the OV section confirmed our findings that non-facilitative transfers exist. Subordinate clauses in German are in OV sequence and the verb is always in the final position of a subordinate clause, whereas subordinate clauses in Chinese and English follow VO sequence. Due to the fact that subordinate clauses with OV sequence were ungrammatical, we expected all participants to correct the sentence by modifying them with Mode 1. However, we found that the German group makes correct judgments in only 48% of the test items, and 45% of them were correctly modified. The English group performs a little bit better in the OV section, with 69% of the teste items were correctly judged and 65% of the them were correctly modified. Thus, we come to the conclusion that both groups were influenced by non-facilitative transfers from their background language German and thought sentences with OV structure were grammatical. The perceived crosslinguistic similarities of abstract structural properties serves as the main reason for language transfers.

Then, are L3 learners affected by different background languages time in third language learning? We found that among both groups, only 5 subjects from the German group made all correct judgments in TC1 section and 2 subjects from the English group scored full points the OV section. The other subjects made incorrect judgments in both TC1 and OV sections, which indicated that they had been affected by non-facilitative transfers from both English and German.

From the perspective of the scores and the reaction time we reconfirmed our conclusions. Although they showed significant differences between the four categories, subjects in the German and English groups performed similarly in the experimental task with no significant differences between the groups. This suggested that their similar background languages (either L1 or L2) simultaneously influenced subjects’ third language learning.

Till now, we have answered the first three research questions posed in the introduction part. We come to the conclusion that (1) both the first and second language are sources of language transfers in TLA. (2) Language transfers do not only occur in one of the background languages that is most similar to the target language. Rather than the similarity of language types, the perceived crosslinguistic similarity of abstract structural properties between languages is the main reason for facilitating language transfers. (3) Language transfers can be both facilitative or non-facilitative.

### 4.2 Language proficiency levels and language transfers in TLA

We first discuss the issue of the relationship between L3 proficiency levels and language transfers in TLA. In order to investigate this issue, the VO structure in subordinate clauses was selected for the design of the experimental materials in this study. As we have mentioned in the methodology part, since the VO structure is the basic principle of sequence in Chinese, we believe that intermediate Chinese learners should have learnt this structure and have a reasonably high level of language proficiency. While the TC structure, on the other hand, has beyond the grammatical points that intermediate Chinese learners should master according to the HSK. Based on this, we artificially created a discrepancy in L3 proficiency levels for each subject group and we assumed that the subjects’ performance in the VO/OV part should be much better than TC part, which is relatively a more difficult structure.

According to the analysis of scores we found that both two groups scored the highest in the VO section, and there was a significant difference with the scores of the other 3 categories. No significant difference between the groups was found. Response time data revealed the same findings that both groups had a shorter response time in the VO part compared with others and there was no significant difference between the groups. As for the findings from sentences modification analysis, we likewise found that the two groups of subjects had the lowest frequency of incorrect judgment in the VO part, although there was still someone who made wrong judgments regarding VO. This phenomenon suggests that although subjects from both groups had acquired the VO structure of Chinese at the intermediate level, they are also affected by the non-facilitative transfers from the background languages. This may be due to the fact that mother tongue and foreign languages learning are different in nature. Bardel and Falk (2007) argued that native and non-native languages were stored in different areas of the brain. They categorized native language learning as procedural memorizing and non-native languages learning as declarative memorizing. Even if L3learners have achieved a high level of L3 language proficiency and already mastered the VO structure, there is still a great possibility of non-facilitative transfers from background languages, which is proved by our findings in both VO and OV sections.

Considering the results above, we conclude that L3 proficiency levels do correlate to language transfers in TLA. As L3 proficiency level increases, the influence of non-facilitative transfers may decrease but will not disappear because of the fundamental differences between foreign language learning and native language learning. As to whether or not learners will still be affected by the non-facilitative transfers of background language when their levels L3 proficiency is high enough or even reaches the level of native speakers, further research and discussion are still needed.

Then we move to the issue in regard to the relationship between proficiency levels of background languages and language transfers in TLA. It has been demonstrated above that these L3 learners of Chinese are affected by the crosslinguistic influence from both L1 and L2 Based on the subjects’ experimental performance we get to know how proficiency levels of background languages affect language transfers.

Since the German group scored significantly higher than the English group in the TC1 category, where German and Chinese have similar structural properties, it can be inferred that subjects of German group were affected by stronger facilitative crosslinguistic influence from German than the English group. Similarly, since the English group scored significantly higher than the German group in the OV section, and both English and Chinese follows VO sequence, it can be inferred that subjects of English group were affected by stronger facilitative crosslinguistic influence from English than the German group. The reaction time data and the sentence correction data showed similar results. Due to the fact that for the German group, the proficiency level of German was higher than that of L2 English and for the English group, the proficiency level of English was higher than that of German, we come to the following conclusion: when there is a similarity in abstract structural properties between the L3 input and the subject’s background languages, the higher the language proficiency level of the background language, the more likely that language transfer will occur. We believe that this conclusion can apply to the multilingual learning with more languages, but further proof is needed.

In summary, we reach the following conclusions: (1) as learners’ L3 proficiency levels increase, the influence of non-facilitative transfers may decrease but may not disappear completely. (2) Background languages with higher proficiency levels are more likely to impose language transfers in TLA.

### 4.3 Implications for teaching and learning Chinese as L3

Firstly, it is found that the perceived similarity of abstract syntactic properties between the background languages and the target language is the main reason for language transfers. From the teachers’ point of view, teachers are the organizers of the teaching process, the guides and facilitators of students’ learning, as well as the researchers of education and teaching, and they should constantly enrich their theoretical reserves and master the relevant knowledge of multilingual learning. They can take the initiative to introduce the theory of third language and multilingual learning in the classroom, helping students to correctly recognize the difference between L1, L2 and L3 learning, and realize the influence of background languages and learning experience in TLA, consciously guiding students to pay attention to the comparison of words, pronunciations and structures between background languages and the target language to promote the facilitative transfers.

Secondly, the study proves the existence of non-facilitative transfers TLA and all background languages are the source of language transfers. From the learners’ point of view, compared with L2 learning, third language learners have more complex language backgrounds. Thus, making mistakes due to non-facilitative transfers is more likely to occur. Taking the two groups of subjects in our study as an example, some learners not only make wrong judgments due to the influence of their mother tongue, but also are affected by non-facilitative transfers from their L2 at the same time. Therefore, learners should not only recognize the inevitability of non- facilitative transfers, understand the special characteristics of TLA, and summarize their mistakes in time, but also consciously draw on previous learning experiences, especially in second language learning, to promote the improvement of language competence.

Thirdly, our study has shown that as L3 proficiency levels increases, learners will gradually be less affected by non-facilitative transfers from the background language. Compared with Indo-European languages, Chinese is unique in that it not only lacks verb inflection, but also differs from other languages in terms of tense and style of expression. Therefore, increasing language input through intensive teaching and practicing is an important way to promote proficiency levels in Chinese. For example, teachers can consciously increase Chinese input in the classroom by rationally designing classroom with traditional Chinese activities and using Chinses as the classroom language. In addition, teachers should pay attention to strengthening learners’ intrinsic motivations and stimulating learners’ enthusiasm, encouraging learners to consciously increase Chinese input outside the classroom by organizing Chinese cultural activities, Chinese talent courses, and introducing Chinese movies and books, etc., so as to consolidate learners’ mastery of the syntactic structure of Chinese in vivid corpus and cultivate the sense of Chinese language and expressions. So that the impact of non-facilitative transfers from the background languages will be minimized.

### 4.4 Conclusions and limitations

We have examined the sources, properties and modes of language transfers in the process of TLA of Chinese by intermediate level German and English native speakers through a behavioral experiment online using Grammaticality Judgment and Correction Task and explored the potential relationship between language proficiency levels and language transfers in this study. Our findings have certain theoretical value and practical significance for the promotion of TLA of Chinese and international Chinese language education.

In this paper, we used the method of computer randomly generating test items to reduce the influence of items order on the experimental results of the subjects. Since we have applied a design crossed by subjects and items, Latin square design is the more traditional and mainstream technique of balancing the order of test items. In future studies, we hope to supplement the Latin square design to confirm our conclusions. Restricted by practical factors, the subjects of this study could have been richer, the selected structures could have been more comprehensive, and the analysis of the problem still has the potential to be in-depth. In the follow-up study, we will continue to improve the experimental design, utilize richer research methods, expand the scope of subjects to further verify the questions and findings of this study to contribute to the promotion of the development of TLA research, as well as to the solution of the actual problems of Chinese learning and teaching as L3 in China.

## Data availability statement

The raw data supporting the conclusions of this article will be made available by the authors, without undue reservation.

## Ethics statement

The studies involving humans were approved by School of Foreign Languages, Renmin University of China. The studies were conducted in accordance with the local legislation and institutional requirements. Written informed consent for participation was not required from the participants or the participants’ legal guardians/next of kin in accordance with the national legislation and institutional requirements.

## Author contributions

ZW: Writing – original draft, Writing – review & editing. CX: Supervision, Writing – review & editing.
